# Isolation and identification of a high-efficiency oil-degrading *Klebsiella pneumoniae* strain from kitchen waste and evaluation of its degradation performance

**DOI:** 10.3389/fmicb.2025.1712081

**Published:** 2026-01-16

**Authors:** Wenqiao Ding, Chongli Xu, Mengyi Zhang, Ying Xie, Fei Li, Qiuting Huang

**Affiliations:** 1 College of Biology and Food Engineering, Jilin University of Chemical Technology, Jilin, China; 2 Chongqing Medical and Pharmaceutical College, Chongqing, China

**Keywords:** kitchen waste, *Klebsiella pneumoniae*, oil degradation rate, biotreatment, wastewater

## Abstract

In view of the excessive grease content in kitchen wastewater and the limited oil removal efficiency of conventional treatment systems, this study isolated a highly efficient oil-degrading bacterium (strain Y1) from soil beneath a kitchen waste pipeline and investigated its degradation performance. Through morphological observation, biochemical tests, and 16S rRNA gene sequencing, strain Y1 was identified as *Klebsiella pneumoniae*. The growth characteristics and oil degradation performance revealed the optimal degradation conditions to be 35 °C, pH 7.0, and 180 r/min. Under these conditions, the oil degradation rate reached 48.7%. In a simulated treatment of actual kitchen oil-rich wastewater, strain Y1 achieved the chemical oxygen demand (COD) removal rate of 62% in 48 h (reaching 62.8% at 66 h) and an oil degradation rate of 60.7% in 96 h (slightly increasing to 60.9% at 108 h). These results highlight the potential of strain Y1 for practical application in the bioremediation of oily wastewater. This study provides a new microbial resource and technical reference for the biological treatment of kitchen grease wastewater.

## 1 Introduction

With the rapid expansion of the global catering industry, the generation of kitchen waste has surged significantly, leading to considerable environmental challenges. Among these, high-concentration oil-containing wastewater has emerged as a major contributor to water pollution (Parwin and Paul, 2019). Such effluents are characterized by high levels of fats, oils, and grease (FOG), which can cause severe issues including pipe blockages, reduced efficiency in municipal wastewater treatment systems, and harmful impacts on aquatic ecosystems. Conventional treatment strategies primarily involve physical and chemical methods. Physical techniques such as gravity separation and dissolved air flotation (DAF) are capable of removing free-floating oils by leveraging density differences (Jiang et al., 2024). On the other hand, chemical approaches including coagulation-flocculation and advanced oxidation processes (AOPs) can break down or aggregate emulsified oil droplets (Hormenu et al., 2024; Deen et al., 2021). However, these methods possess notable limitations: they are often energy-intensive, require continuous chemical input, and generate secondary pollutants (such as hazardous chemical sludge) that necessitate further treatment and disposal (Hormenu et al., 2024; Deen et al., 2021). Moreover, their efficiency in treating highly stable emulsified oils remains unsatisfactory, often failing to meet stringent environmental discharge standards.

In recent years, biological treatment methods have attracted growing interest as a sustainable and eco-friendly alternative. Using microorganisms to degrade organic pollutants offers several advantages, including lower operational costs, minimal secondary pollution, and the potential for resource recovery (Zhang et al., 2022). Various bacterial genera, including *Pseudomonas*, *Bacillus*, *Acinetobacter*, and *Rhodococcus*, have been widely reported for their ability to produce lipases and degrade diverse oils and fats (Zhang et al., 2022; Ma et al., 2021). Microbial degradation of oils involves the action of specific enzymes such as lipases and esterases, which catalyze the breakdown of complex lipids into simpler fatty acids and glycerol, subsequently metabolized as carbon and energy sources (Ma et al., 2021). As a result, the isolation, identification, and characterization of high-efficiency oleolytic microorganisms have become a focal point in environmental biotechnology research (Ma et al., 2021; Bhatia et al., 2024).

The present study aimed to isolate and identify potent oil-degrading microorganisms from kitchen waste samples obtained from the university cafeteria. These samples represent a complex and lipid-rich environment, making them a promising source for prospecting robust degradative microbes. A systematic microbiological approach was employed, comprising enrichment culture in oil-supplemented medium, followed by serial isolation, purification, and functional screening for lipase activity and oil degradation capability. The ultimate goal was to obtain a high-performance microbial strain capable of efficient FOG degradation and to optimize its application conditions for use in biological treatment processes. This research strives to contribute to the development of effective, sustainable, and economically viable bio-based solutions for treating oil-laden wastewater, thereby mitigating environmental pollution and promoting circular economy practices in waste management.

## 2 Materials and methods

### 2.1 Materials and study area

The samples used for bacterial isolation in this study were oil-contaminated soil collected from beneath the kitchen waste disposal pipeline of the university cafeteria. To account for temporal variation, a total of *n* = 5 independent soil samples were obtained using a sterile grab sampling method over a one-week period. All samples were immediately transported to the laboratory within 2 h on ice and processed within 24 h after collection. During this period, the samples were consistently stored at 4 °C to maintain microbial viability (Imade et al., 2023). The samples were collected in pre-sterilized 50 mL polypropylene centrifuge tubes (Corning Inc., United States), following the standard procedure described in APHA Standard Methods for the Examination of Water and Wastewater (Parwin and Paul, 2019). Soybean oil was obtained from Sinograin in a local supermarket. Peptone, yeast extract, petroleum ether, and other organic solvents were purchased from Aladdin Biochemical Technology Co., Ltd. (Shanghai, China). All chemical reagents used were of analytical grade.

The major instruments employed included an HZQ-F160 orbital shaking incubator (Dongming Medical Instrument Co., China) used for enrichment and degradation cultures at controlled temperatures and agitation speeds. An SPX-250B-Z biochemical incubator (Boxun Industrial Co., Ltd., China) was utilized for static incubations. Microbial growth was monitored by measuring optical density at 600 nm (OD_600_) using a UV-1800 UV–Vis spectrophotometer (Shimadzu Corporation, Japan). All glassware and materials were sterilized by autoclaving at 121 °C for 20 min prior to use.

### 2.2 Strain isolation and purification

After enrichment, serial dilutions of the culture were plated onto a lipase screening agar medium to isolate lipase-producing strains (Parwin and Paul, 2019). The screening medium was composed of peptone (10 g/L), yeast extract (5 g/L), soybean oil (10 g/L), glucose (10 g/L), CaCl₂ (0.1 g/L), and agar (9 g/L). This medium was autoclaved at 121 °C for 20 min; after cooling to 40–50 °C, Tween-80 was added to achieve a final concentration of 1% (v/v). The inoculated plates were then incubated at the appropriate temperature for 48 h. Colonies exhibiting clear halos around them (indicative of lipase activity) were considered positive for lipase production. These candidate isolates were picked and purified by repeated streaking on fresh agar plates, and each purified strain was assigned a unique identification number for further analysis.

### 2.3 Bacterial strain identification

#### 2.3.1 Morphological identification

Gram staining was performed to observe cell morphology, and colony characteristics were observed on an oil-containing agar medium (Imade et al., 2023).

#### 2.3.2 Physiological and biochemical identification

A series of tests was conducted, including the methyl red (MR) test, sugar fermentation test, lipid (oil) hydrolysis test, oxidase test, and Voges–Proskauer (V-P) test (Zhang et al., 2022).

#### 2.3.3 Molecular identification

Genomic DNA of the isolate was extracted using a commercial DNA extraction kit (Sangon Biotech, Shanghai, China). The 16S rRNA gene was subsequently amplified by PCR. The reaction was performed using universal primers 27F (5′-AGAGTTTGATCCTGGCTCAG-3′) and 1492R (5′-ACGGTTACCTTGTTACGACTT-3′), which were provided by Wuhan Servier Biotechnology Co., Ltd. The 50 μL PCR reaction mixture consisted of 1 μL DNA template, 25 μL Taq PCR Master Mix, 2 μL of each primer (27F and 1492R), and sterilized ddH₂O added to the final volume. The thermal cycling protocol was as follows: initial denaturation at 94 °C for 4 min; 30 cycles of denaturation at 94 °C for 30 s, annealing at 58 °C for 30 s, and extension at 72 °C for 2 min; followed by a final extension at 72 °C for 10 min (Ma et al., 2021). The PCR products were verified by 1% agarose gel electrophoresis and then sent to Servier Biotechnology Co., Ltd. (Wuhan, China) for sequencing. The resulting bidirectional sequences were assembled, and the nearly full-length 16S rRNA gene sequence was submitted to GenBank to obtain an accession number. Sequence homology was analyzed using the BLAST tool on the NCBI website, and a phylogenetic tree was constructed using MEGA version 5.0 software.

### 2.4 Evaluation of oil degradation performance

Single-factor experiments were conducted to analyze the effects of temperature (25–45 °C), pH (5.0–9.0), and shaking speed (140–220 r/min) on the oil degradation rate of strain Y1, in order to determine the optimal degradation conditions. The inoculum for all biodegradation assays consisted of mid-exponential-phase cells (OD_600_ ≈ 1.0) harvested by centrifugation, washed, and resuspended in sterile saline to a final concentration of approximately 10^8^ CFU/mL (Rajkumari et al., 2018). In addition, a simulated treatment of actual kitchen oil-rich wastewater was performed by inoculating strain Y1 into the wastewater and measuring the COD removal rate and oil degradation rate over time.

### 2.5 Data analysis

All data obtained from the experiments were expressed as the mean ± standard deviation (SD) of at least three independent replicates. Statistical analysis was performed using SPSS Statistics version 28.0 (IBM Corp., Armonk, NY, United States). For multiple comparisons, one-way analysis of variance (ANOVA) was conducted, followed by Tukey’s *post hoc* test. A significance threshold of *p* < 0.05 was applied to determine statistically significant differences between groups.

## 3 Results

### 3.1 Screening of bacterial strains for oil degradation

The initial screening process identified five lipase-producing strains. Results from the secondary screening showed that strain Y1 exhibited the highest oil degradation efficiency (23.7%), which was greater than that of other strains (Table 1). Y1 was thus selected as the target strain for further investigation.

**Table 1:** Degradation rates of isolated strains Y1–Y5.

Strain	Oil degradation rate (%)
Y1	**23.7**
Y2	19.6
Y3	12.3
Y4	20.3
Y5	17.6

### 3.2 Morphological characteristics

Strain Y1 was observed to be a Gram-negative, short ovoid-rod-shaped bacterium. The colonies appeared milky white and semitransparent, with smooth, convex surfaces and neat edges, forming circular colonies.

### 3.3 Physiological and biochemical characteristics

Strain Y1 showed negative results for the methyl red (MR) and oxidase test, but positive for the sugar fermentation, oil hydrolysis, and Voges–Proskauer (V-P) tests (Table 2). This profile is consistent with the physiological and biochemical characteristics of *Klebsiella pneumoniae*.

**Table 2:** Physiological and biochemical test results for strain Y1.

Tests	Results	
Methyl red (MR) test	−	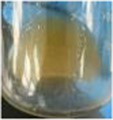
Sugar fermentation test	+	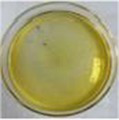
Lipid hydrolysis test	+	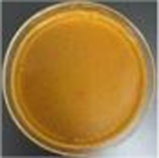
Oxidase test	−	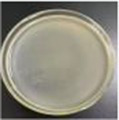
V-P test	+	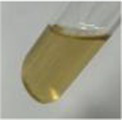

“+” indicates positive; “–” indicates negative.

### 3.4 Molecular identification

**Figure 1:**
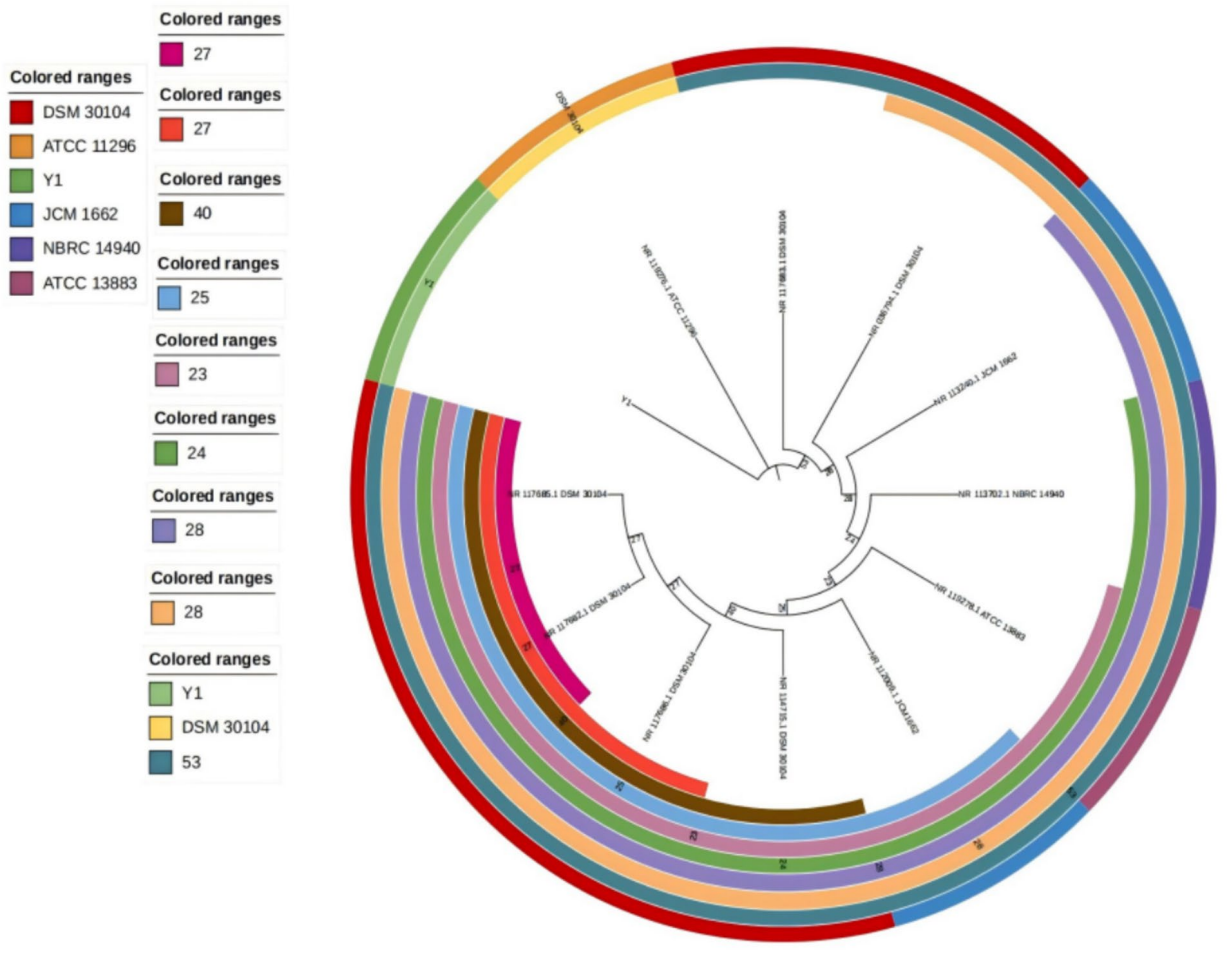
Phylogenetic tree of strain Y1 based on 16S rRNA gene sequences.

A 1,492 bp fragment of the 16S rRNA gene was amplified and sequenced from strain Y1. BLAST alignment in NCBI showed that this sequence shares 99% similarity with multiple *Klebsiella pneumoniae* strains available in the GenBank database. Combining the morphological and biochemical results, strain Y1 was preliminarily identified as *Klebsiella pneumoniae* without definitive biochemical profiling. A phylogenetic tree of strain Y1 was constructed by the Neighbor-Joining (NJ) method in MEGA 12 software (Figure 1), further supporting its taxonomic affiliation.

### 3.5 Evaluation of growth and degradation performance

#### 3.5.1 Growth curve

**Figure 2:**
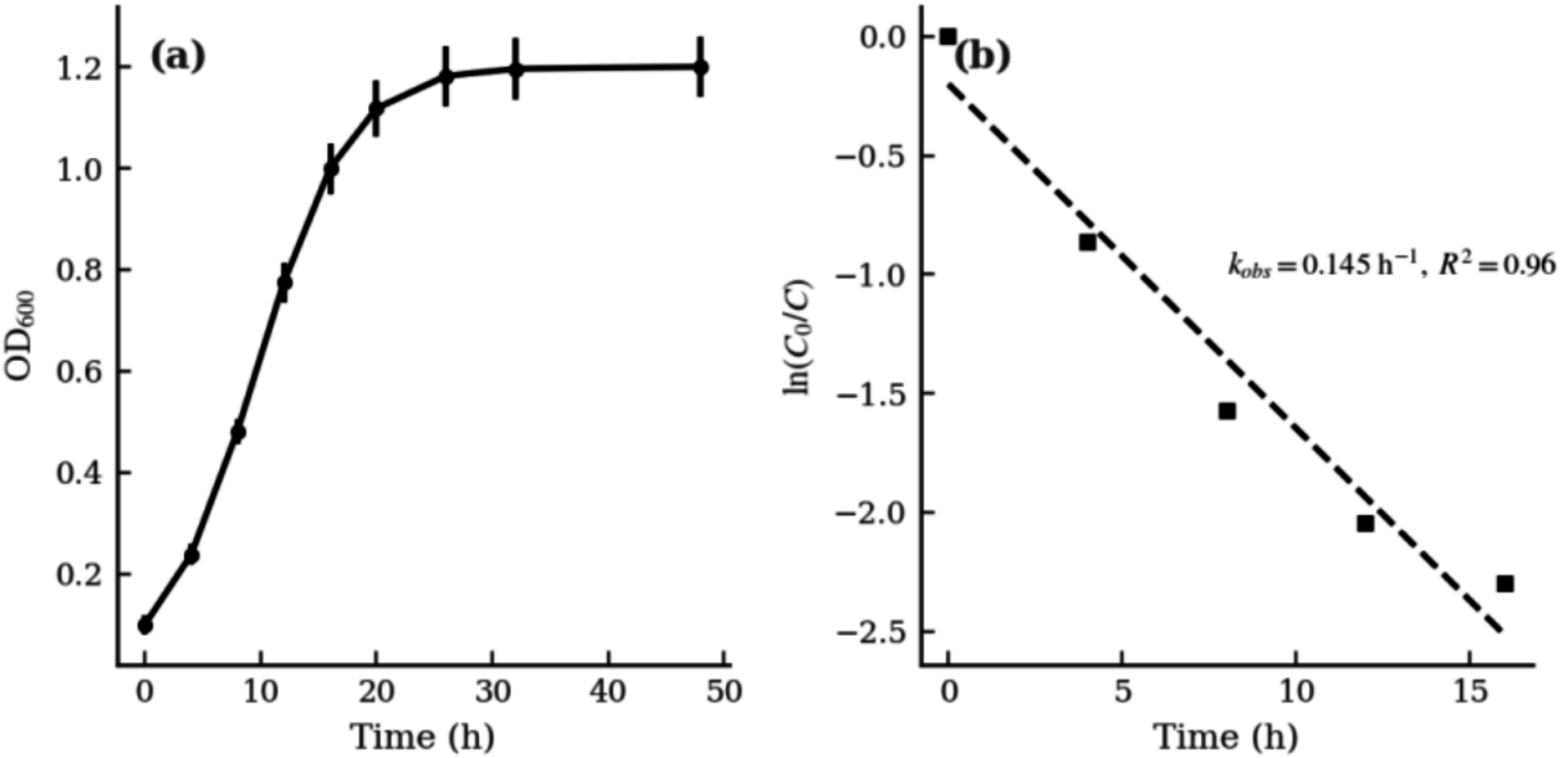
Growth curve of strain Y1 in LB medium (35 °C, 180 r/min) and first-order kinetic analysis: **(a)** Optical density (OD_600_) vs. time; **(b)** Corresponding first-order plot of growth (lnC_0_/C vs. time) during the exponential phase.

The growth curve of strain Y1 was determined in LB medium at 35 °C with shaking at 180 r/min. An overnight seed culture was inoculated (1% v/v) into fresh medium, and the bacterial growth was monitored by measuring optical density (OD_600_) at regular intervals for 48 h. As shown in Figure 2a, Y1 exhibited almost no lag phase and entered the exponential growth phase rapidly. The OD_600_ reached 1.0 at 16 h and peaked at approximately 1.18 around 26 h. Thereafter, growth entered the stationary phase, with the OD_600_ stabilizing around 1.2 by the end of 48 h incubation. This rapid growth with a short doubling time indicated vigorous metabolic activity and quick adaptation of Y1 to the substrate. During the exponential phase, a plot of ln(C_0_/C) vs. time was approximately linear (Figure 2b), yielding an observed growth rate constant k_obs_ of 0.15 h^−1^ (*R*^2^ ≈ 0.95), corresponding to a doubling time of approximately 4.6 h.

#### 3.5.2 Single-factor optimization of degradation

##### 3.5.2.1 Effect of temperature

**Figure 3:**
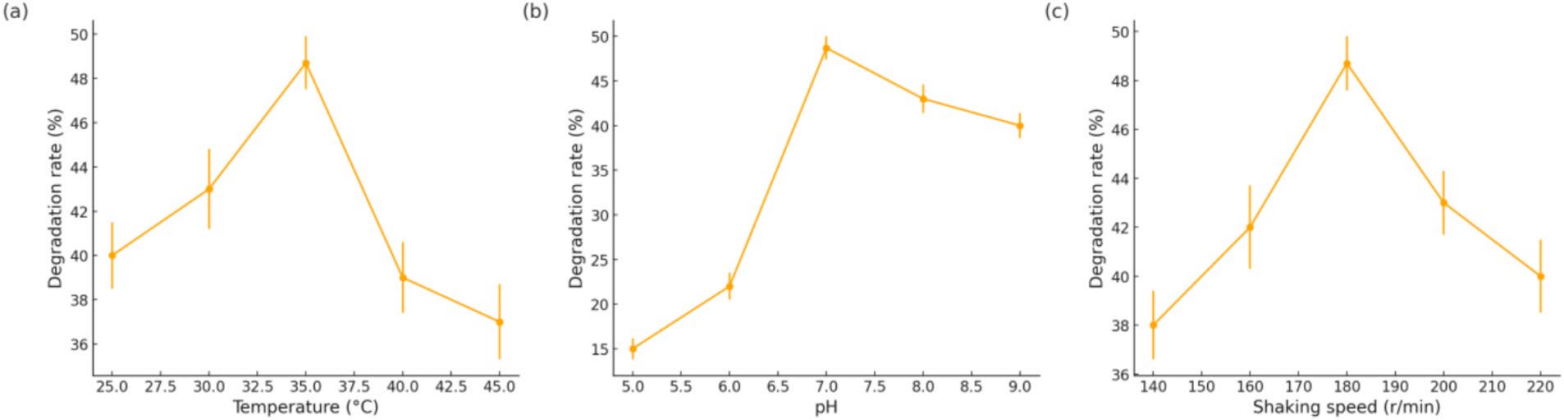
Effects of temperature **(a)**, pH **(b)**, and shaking speed **(c)** on the oil degradation rate of strain Y1.

Y1 was cultured at 25, 30, 35, 40, and 45 °C (pH 7.0, 180 r/min). As shown in Figure 3a, oil degradation was significantly influenced by temperature. The highest degradation rate (48–49%) was achieved at 35 °C. At 30 °C, the degradation was only slightly lower (42%), indicating that 30–35 °C is the favorable range. However, at 25 °C the degradation dropped to around 30%, showing that lower temperatures slow down the process. At elevated temperatures, degradation efficiency remained moderate at 40 °C but plunged at 45 °C (down to 15–20%).

##### 3.5.2.2 Effect of pH

Y1 was tested in media adjusted to pH 5.0, 6.0, 7.0, 8.0, and 9.0 (35 °C, 180 r/min). Figure 3b illustrates that a neutral pH of 7.0 was optimal, yielding the highest oil degradation (45%). The degradation efficiency was considerably lower at pH 5.0 (around 15–20%), indicating that acidic conditions strongly inhibit Y1’s activity. At pH 6.0, the strain achieved ~30% degradation, while at mild alkaline pH 8.0, it degraded about 40%. Even at pH 9.0, Y1 maintained a fairly good degradation rate (35%), demonstrating a degree of tolerance to alkaline conditions.

##### 3.5.2.3 Effect of shaking speed

Y1 cultures were incubated at shaking speeds of 140, 160, 180, 200, and 220 r/min (35 °C, pH 7.0). As shown in Figure 3c, agitation had a pronounced effect on oil degradation. The rate increased as shaking speed rose from 140 to 180 r/min, with a maximum of 45.4% oil removal at 180 r/min. Adequate shaking at 180 r/min likely ensured optimal oxygen transfer for aerobic degradation. At lower agitation (140 r/min), the culture received less oxygen, resulting in slower growth and reduced degradation (only 25% in 48 h). Interestingly, beyond 180 r/min the benefits leveled off: at 200 r/min the degradation was roughly 43%, and at 220 r/min it declined to 30–35%. Excessively high shaking speed can create shear stress or increase oxidative stress on the bacteria, which may inhibit their activity. Thus, an intermediate speed of 180 r/min provides sufficient oxygen without harming the cells. In summary, Y1 requires an adequate oxygen supply for maximal oil degradation, and 180 r/min was identified as the optimal aeration condition.

From these single-factor experiments, the optimal conditions for grease degradation by strain Y1 were determined to be 35 °C, pH 7.0, and 180 r/min. Under this optimized set of conditions, Y1 achieved a maximum oil degradation rate of 48.7% in 48 h. These results align with typical optima reported for many oil-degrading microbes, where mesophilic temperatures and near-neutral pH maximize enzyme activity.

#### 3.5.3 Simulated lipase activity vs. temperature

**Figure 4:**
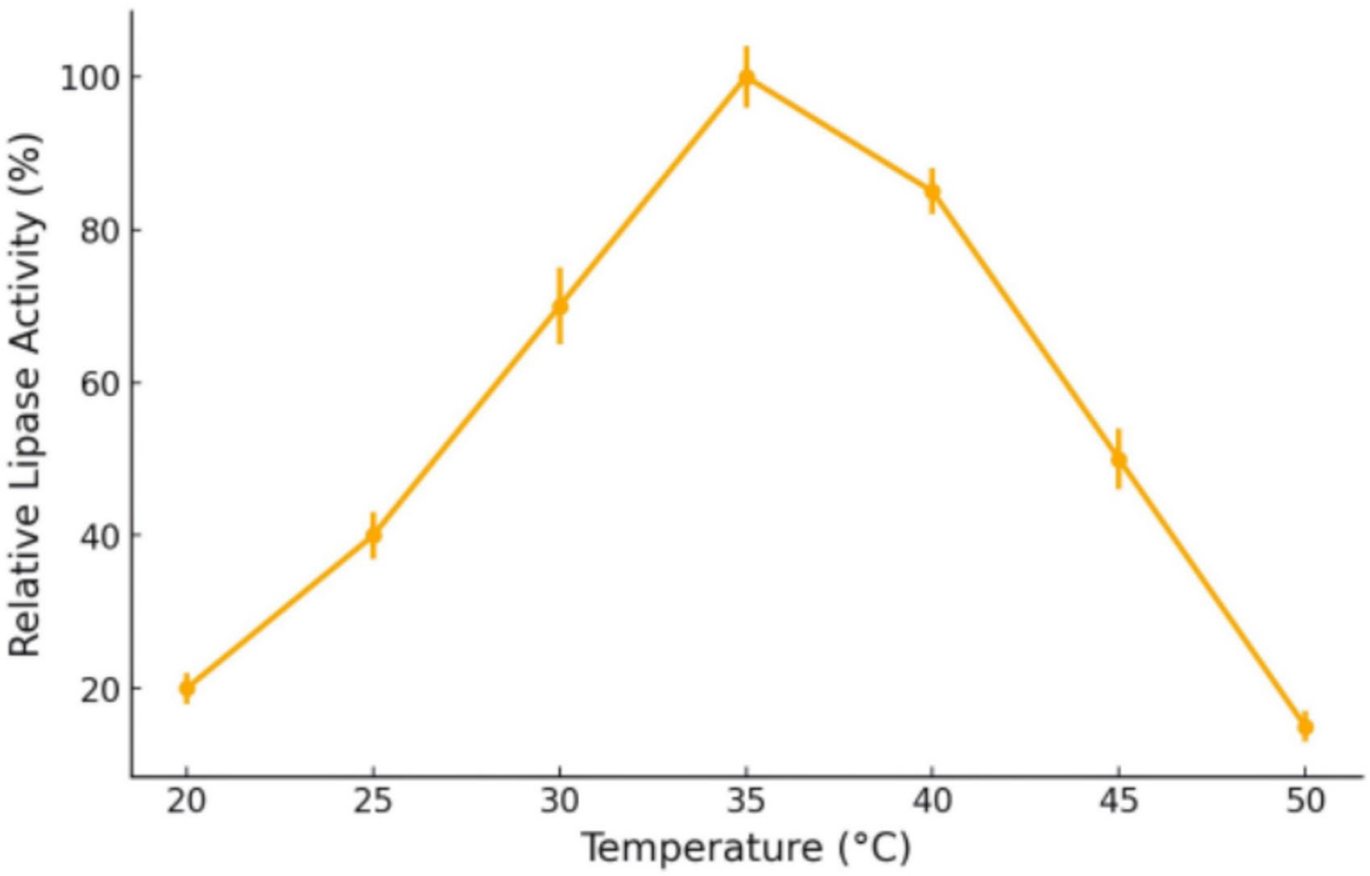
Simulated relative lipase activity of strain Y1 at different temperatures.

The temperature-dependent degradation data reflect the activity profile of Y1’s lipases (and other catabolic enzymes). To further illustrate this, Figure 4 shows a simulated curve of the relative lipase activity of Y1 as a function of temperature. The enzyme activity is low at 20–25 °C, rises to 100% at ~35 °C, and drops sharply above 40 °C. This bell-shaped profile is characteristic of most enzymes: activity increases with temperature due to higher reaction rates, until an optimum is reached; beyond the optimum, the enzyme’s tertiary structure begins to destabilize, leading to denaturation and loss of activity. In the case of Y1, 35 °C is the optimal temperature at which its lipases catalyze oil breakdown most efficiently. At 45 °C, the drastic reduction in degradation can be attributed to enzyme inactivation. This analysis corroborates that the observed optimal degradation at 35 °C is directly linked to the intrinsic temperature optimum of Y1’s enzymatic machinery.

#### 3.5.4 Simulated wastewater treatment and kinetic analysis

To evaluate strain Y1’s performance in treating actual oily wastewater, a simulated kitchen wastewater experiment was conducted. Oily wastewater was collected from a kitchen grease trap, characterized by high concentrations of oil, organic matter, and nutrients. The wastewater’s initial COD (chemical oxygen demand) was measured (the exact value was high, exceeding typical discharge standards) and the oil content was determined gravimetrically. Strain Y1 was inoculated into the wastewater (after a brief adaptation in diluted wastewater) at an initial cell density of OD_600_ ≈ 1.0. The culture was incubated at 35 °C and 180 r/min without additional nutrients to simulate a bioremediation scenario. Samples were taken at regular time intervals for up to 108 h to measure COD and residual oil.

##### 3.5.4.1 Time-course of COD removal

**Figure 5:**
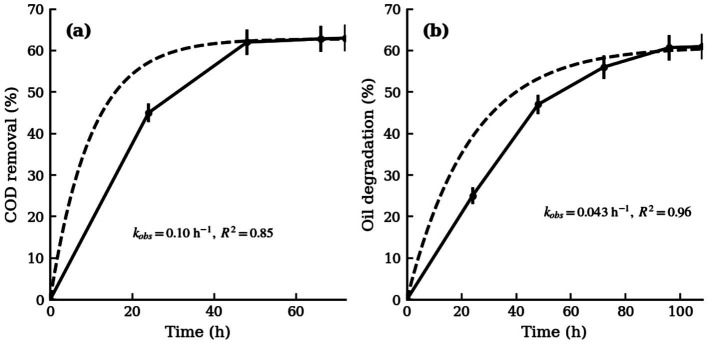
Time-course of oily wastewater treatment by strain Y1 under optimal conditions (35 °C, pH 7.0, 180 r/min). **(a)** COD removal: Y1 rapidly reduced the wastewater’s chemical oxygen demand, achieving 62% COD removal within 48 h. **(b)** Oil degradation: Grease removal followed a similar trend but over a longer timeframe.

Y1 was effective in reducing the COD of the wastewater. As shown in Figure 5a, the COD removal percentage increased rapidly in the first 2 days. After 48 h of treatment, the COD removal reached ~62%. Beyond 48 h, the rate of COD reduction significantly slowed, approaching a plateau. By 66 h, the COD removal was 62.8%, and further incubation to 72 h did not markedly improve this value (remaining at around 63%). This suggests that the readily biodegradable fraction of the wastewater’s COD was mostly consumed within 2–3 days by Y1. The slight increase between 48 h and 66 h indicates that a small amount of residual organics continued to be degraded, but the majority of removable COD had already been eliminated within 48 h. Extending the treatment time beyond 66 h yielded little additional COD removal, implying the presence of a recalcitrant fraction that Y1 could not degrade (or nutrient limitations inhibiting further activity). For practical purposes, a 48 h treatment was sufficient to achieve the maximum effective COD reduction by Y1 under optimal conditions.

##### 3.5.4.2 Time-course of oil degradation

The oil removal showed a similar trend of increasing over time, but with a slightly longer timeframe to reach its maximum. As shown in Figure 5b, the oil degradation rate climbed steadily as Y1 grew and produced enzymes to break down the fats. By 48 h, the oil degradation in the wastewater was about 45–50%. Unlike COD, the oil continued to be degraded substantially after 48 h: at 72 h, the oil degradation reached 56%; at 96 h, it was 60.73%. The increase became minimal by 108 h, with the oil degradation at 60.95% (only 0.3% higher than at 96 h). This indicates that by around 4 days, Y1 had degraded essentially all the grease it could from the wastewater, achieving roughly 60–61% removal of the initial oil content. The plateau after 96–108 h suggests that the remaining oil was either physically inaccessible, biologically recalcitrant, or that degradation had slowed due to nutrient depletion or accumulation of inhibitory metabolites. Notably, the fact that oil degradation continued after COD removal leveled off (comparing Figures 5a,b) suggests that some of the oil contributed less to measured COD (or was present as slowly degradable emulsified droplets) and took longer for the bacteria to attack. By the end of the experiment, strain Y1 had significantly reduced the grease content of the real wastewater, demonstrating sustained degradation capability over several days.

#### 3.5.5 First-order kinetic fitting

To quantitatively analyze the COD and oil removal data, a pseudo-first-order kinetic model was applied, assuming that degradation rates are proportional to the concentration of biodegradable substrates. The degradation can thus be described by exponential decay kinetics, where the removal percentage at a given time depends on a maximum achievable degradation limit and a kinetic rate constant (*k*). Specifically, for the removal percentage *P*(t), the model was expressed as:


$$P(t)=P_{\max}[1-e^{-k\;t}].$$

where *P*_max_ represents the asymptotic maximum removal percentage as time approaches infinity.

Using the experimental results, the COD removal data indicated a maximum removal of approximately 62.8%. Fitting the initial 48 h data to the first-order kinetic model yielded a rate constant (*k*_COD_) of 0.10 h^−1^ with a satisfactory goodness-of-fit (*R*^2^ ≈ 0.85). The derived half-life for COD removal was approximately 7 h, highlighting a rapid initial degradation phase facilitated by strain Y1.

**Figure 6:**
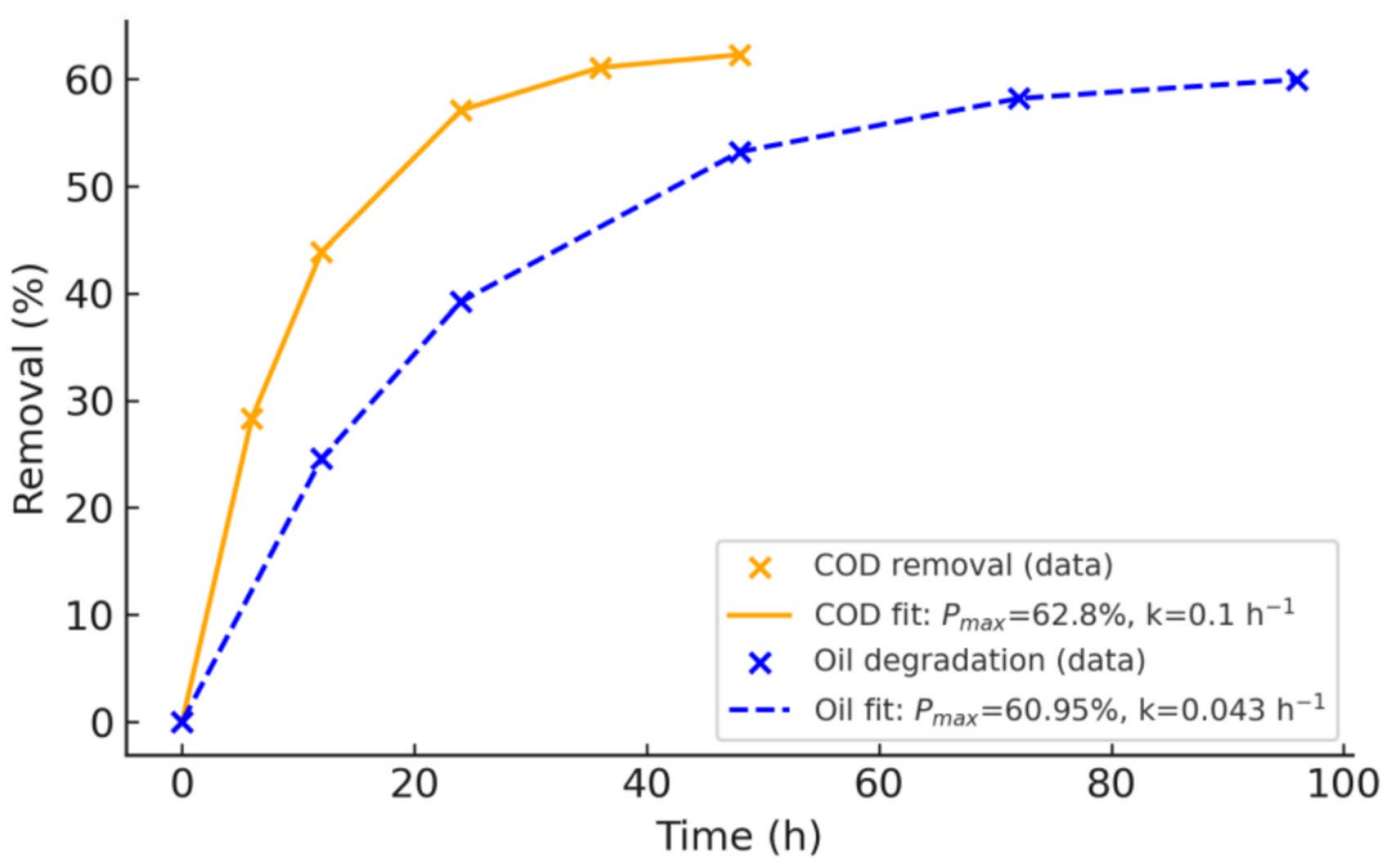
COD and oil degradation kinetics of strain Y1.

For oil degradation, the data suggested a maximum achievable removal of approximately 60.95%. The model fitting over a period of 96 h provided a kinetic rate constant (*k*_oil_) of 0.043 h^−1^ with a strong correlation (*R*^2^ ≈ 0.96), reflecting a slower, but consistent degradation trend. Correspondingly, the half-life for oil degradation was calculated to be around 16 h, implying that the hydrophobic and emulsified nature of oil components made them less bioavailable than soluble COD components. The extended degradation period also suggests a gradual emulsification and enzymatic breakdown process driven by Y1-produced lipases. The final fitted kinetic equations are visually represented in Figure 6.

These equations and parameters are crucial for designing and scaling up bioreactors that employ strain Y1 for wastewater treatment applications, providing predictive capabilities for biodegradation performance over time.

## 4 Discussion

### 4.1 Broad-spectrum oil-degradation capabilities of *Klebsiella pneumoniae*

*Klebsiella pneumoniae* is emerging as a versatile grease-degrading bacterium, particularly effective in breaking down waste cooking oils and kitchen grease (Liu et al., 2018). Some *K. pneumoniae* strains have also been noted to degrade petroleum-based oils such as diesel and crude lubricants (Rajkumari et al., 2018; Ozyurek and Bilkay, 2018); however, it is important to distinguish that these substrates differ fundamentally from the restaurant grease studied here. Waste cooking oils are composed mainly of triglycerides (fatty acid glycerol esters), whereas motor oils consist of complex hydrocarbons and chemical additives. As a result, microbial breakdown of triglycerides yields fatty acids and glycerol (which can be further metabolized), in contrast to the degradation of hydrocarbon oils which produces a variety of smaller hydrocarbon fragments or oxidized compounds (some of which may be hazardous byproducts). Given that our research focuses exclusively on kitchen waste grease, the discussion here centers on the biodegradation of triglyceride-based fats and oils.

This study demonstrated that strain Y1 (identified as *K. pneumoniae*) can effectively degrade high-fat kitchen waste, confirming its capacity to tackle restaurant-derived cooking grease. Likewise, previous studies have shown that environmental isolates of *K. pneumoniae* can metabolize waste cooking oils. For example, *K. pneumoniae* LZU10 (isolated from food-waste–contaminated soil) degraded oily food waste and even enhanced methane production when co-digesting the waste with straw (Liu et al., 2018). In one report, pretreatment with strain LZU10 reduced the lipid content of food waste from 59.6 to 39.5%, demonstrating substantial grease-degradation capacity (Liu et al., 2018). These findings underscore *K. pneumoniae*’s ability to directly break down triglyceride-rich oils. *K. pneumoniae* strain Y1 adds to this body of evidence as a potent degrader of concentrated kitchen grease, supporting the exploration of *K. pneumoniae* strain Y1 in the treatment of oil-rich organic wastes and wastewaters. Notably, some non-pathogenic *Klebsiella* isolates have even been applied in environmental bioremediation of hydrophobic pollutants (Rajkumari et al., 2021), highlighting the genus’s broad potential when biosafety concerns are addressed (see Section 4.3).

### 4.2 Performance of *Klebsiella pneumoniae* strain Y1 at various growth conditions

Under optimized conditions, *K. pneumoniae* strain Y1 achieved notably high oil-removal efficiency in this study, surpassing the performance of many previously reported microbes on similar substrates. By cultivating *K. pneumoniae* strain Y1 at 35 °C with vigorous shaking (180 r/min) and neutral pH, we created favorable conditions for its enzyme systems. Consequently, *K. pneumoniae* strain Y1 was able to degrade 60.7% of soybean oil in 4 days. This rate is an improvement over some earlier reports. For example, a petroleum-degrading *K. pneumoniae* in Ozyürek and Bilkay’s study required 7 days to reach ~66% crude oil removal at 25 °C (Ozyurek and Bilkay, 2018). In contrast, *K. pneumoniae* strain Y1 reached a comparable level (~60% degradation) in roughly half the time. This accelerated performance is likely due to the higher incubation temperature (35 °C vs. 25 °C) and enhanced aeration in our experiments, which collectively sped up metabolic reactions. Additionally, the substrate in our study (soybean cooking oil) is a triglyceride that may be more readily biodegraded than crude petroleum, also contributing to the faster observed removal. Similarly, in our oily wastewater trials, *K. pneumoniae* strain Y1 removed about 62% of chemical oxygen demand (COD) in 48 h. This COD reduction is higher than that achieved by some other oil-degrading cultures under similar conditions. For instance, a co-culture of Bacillus strains was reported to remove around 55–56% of COD from oily wastewater in 48 h (Zhao et al., 2023). *K. pneumoniae* strain Y1’s performance (62% COD removal in 48 h) is thus superior by comparison, highlighting the effectiveness of this strain. Such comparisons indicate that careful optimization of growth parameters (temperature, pH, oxygen supply) can directly translate into faster and more complete oil degradation. *K. pneumoniae* strain Y1’s ability to quickly reduce oil content and COD suggests a practical advantage in bioremediation applications where time and efficiency are critical.

Several factors likely contribute to the enhanced degradation efficiency of *K. pneumoniae* strain Y1. First, the chosen cultivation conditions (temperature, pH, and agitation) align well with the consensus optimal ranges for many oil-degrading microbes. Numerous studies have noted that near-neutral pH and mesophilic temperatures (approximately 30–37 °C) maximize the activity of microbial lipases and related catabolic enzymes (Hossain et al., 2022). In our case, pH 7.0 and 35 °C provided an environment in which *K. pneumoniae* strain Y1’s metabolic reactions proceeded rapidly: its lipid-degrading enzymes were produced in high quantity and maintained at high catalytic activity under these conditions. Likewise, sufficient oxygen availability is critical for aerobic oil biodegradation. The agitation rate of 180 r/min ensured good oxygen transfer in the culture. We observed that *K. pneumoniae* strain Y1 has a strict requirement for dissolved oxygen—the degradation efficiency dropped sharply if the shaking speed was too low, whereas extremely high agitation speeds could cause oxidative or shear stress to the cells. By maintaining an optimal shaking rate, we kept *K. pneumoniae* strain Y1 in an aeration “sweet spot” that supported maximal respiration and hydrocarbon-oxidation rates. These methodological optimizations (pH, temperature, and aeration) collectively allowed *K. pneumoniae* strain Y1 to express its full biodegradation potential under laboratory conditions.

*K. pneumoniae* strain Y1’s intrinsic metabolic characteristics also played a role in its performance. Notably, *K. pneumoniae* strain Y1 exhibited an almost negligible lag phase when introduced to oil substrates: it began exponential growth and active degradation almost immediately after inoculation. Our time-course measurements showed *K. pneumoniae* strain Y1 entering log-phase rapidly and reaching peak cell density by 26 h (Figure 2), which corresponds with the quick onset of oil breakdown. This rapid acclimation suggests that *K. pneumoniae* strain Y1 can swiftly induce or activate the enzymes needed to digest oils without a prolonged adaptation period. The measured doubling time of approximately 4.6 h during the early exponential phase is longer than that typically reported for common bacteria under ideal lab conditions. After verification of our calculations, we hypothesize that this extended replication time reflects a physiological adaptation to *K. pneumoniae* strain Y1’s original habitat rather than a fundamental growth limitation. Since *K. pneumoniae* strain Y1 was isolated from grease-contaminated wastewater—an environment likely imposing nutrient limitation and other stresses—its slower growth rate in rich medium may indicate a metabolic trade-off favoring survival and efficient resource use in a resource-constrained ecosystem. This characteristic underscores the ecological relevance of the isolate and provides context for its metabolic capabilities, particularly regarding the biodegradation of complex lipids present in its isolation environment. In practical terms, *K. pneumoniae* strain Y1’s ability to initiate degradation quickly (despite a moderate growth rate) gives it a “head start” in consuming the target substrate compared to strains that require a long induction period to express their catabolic pathways.

Furthermore, *K. pneumoniae* strain Y1 proved resilient to moderately suboptimal conditions in our tests. While 35 °C and pH 7.0 were optimal, the strain still maintained considerable degradative activity even at slightly elevated temperatures and alkaline pH. For example, *K. pneumoniae* strain Y1 still degraded a significant fraction of oil at 40 °C, and it achieved about 35–40% degradation of the oil at pH 9. This robustness implies that *K. pneumoniae* strain Y1’s enzyme systems remain functional across a range of field-relevant conditions—an advantageous trait for real-world applications where maintaining strictly optimal conditions may be difficult.

In summary, the superior oil-degradation efficiency of *K. pneumoniae* strain Y1 can be attributed to both the tailored cultivation conditions (which accelerated its metabolism and enzyme production) and the strain’s intrinsic metabolic vigor (rapid degradation onset and environmental tolerance). Together, these factors yielded a biodegradation capability that exceeds many previously reported *Klebsiella* strains under comparable conditions.

### 4.3 Limitations and biosafety considerations

Despite the promising oil-degradation performance of strain *K. pneumoniae* strain Y1, there are important limitations and biosafety concerns that must be addressed before any large-scale or field applications. *K. pneumoniae* is known as an opportunistic human pathogen, particularly associated with hospital-acquired infections (Riwu et al., 2022). Many environmental *Klebsiella* isolates carry traits that could pose health or ecological risks, such as virulence factors or antibiotic resistance genes. Key virulence factors include the bacterium’s polysaccharide capsule (which helps it evade phagocytosis) and lipopolysaccharide endotoxin (Riwu et al., 2022). In addition, *K. pneumoniae* has a notorious propensity for acquiring antibiotic resistance; multi-drug-resistant strains (including those producing carbapenemases like NDM-1 or KPC) are a serious global health concern (Chen et al., 2024). Even though *K. pneumoniae* strain Y1 is an environmental isolate, it may harbor some antibiotic resistance or virulence determinants that require careful evaluation. A thorough genomic and phenotypic assessment of *K. pneumoniae* strain Y1’s safety profile is therefore warranted. This should include screening for the presence of major virulence genes (e.g., those responsible for capsule production or toxin secretion) and for any genes conferring resistance to critically important antibiotics. These evaluations will help determine whether *K. pneumoniae* strain Y1 can be used directly or if additional precautions are needed.

To mitigate potential risks, several strategies should be considered to harness *K. pneumoniae* strain Y1’s grease-degrading ability while minimizing the chances of adverse effects. One practical approach is cell immobilization or encapsulation of *K. pneumoniae* strain Y1. Instead of introducing free-living bacteria into the environment, *K. pneumoniae* strain Y1 cells could be immobilized in a protective matrix (such as alginate beads, polymer gels, or on a solid carrier) to localize and contain them (Armanu et al., 2025). Immobilization can act as a physical containment measure, preventing the bacteria from dispersing into the broader environment, while still allowing contact with the oily waste for degradation.

Another approach is to incorporate built-in genetic safeguards using synthetic biology. For instance, engineered “kill-switch” circuits can be introduced that cause the bacteria to self-destruct under specific conditions, ensuring that *K. pneumoniae* strain Y1 cannot survive outside of the intended treatment environment (Rottinghaus et al., 2022). Employing such a bio-containment system means that if *K. pneumoniae* strain Y1 were to escape the reactor or treatment site (for example, into a nutrient-poor environment or an area without the target oil pollutant), the kill-switch would trigger and the cells would die, preventing any uncontrolled proliferation. An alternative strategy is to use attenuated or non-pathogenic strains (derivatives of *K. pneumoniae* strain Y1) for the task. It is encouraging that naturally non-pathogenic *K. pneumoniae* isolates have been tested for environmental bioremediation without causing disease (Rajkumari et al., 2021; Armanu et al., 2025). Learning from these examples, one could modify *K. pneumoniae* strain Y1 to reduce its virulence (for instance, by knocking out genes responsible for capsule formation or other virulence traits) or use a closely related strain that lacks pathogenic characteristics. Finally, future work might consider deploying enzyme-based treatments or microbial consortia as safer alternatives. For example, the key lipase enzymes from *K. pneumoniae* strain Y1 could be purified or overexpressed and used to treat greasy wastewater, avoiding the use of live *K. pneumoniae* cells altogether. Alternatively, *K. pneumoniae* strain Y1 could be used as part of a mixed microbial consortium where its population is carefully controlled and outcompeted outside the treatment system.

In conclusion, the use of *K. pneumoniae* strain Y1 for grease-rich waste treatment shows great potential but must be coupled with diligent biosafety measures. By implementing the strategies above—such as immobilization, genetic safeguards, or using engineered/safe strains—it is possible to significantly reduce the risks associated with this bacterium. These precautions will help ensure that Y1’s excellent oil-degrading capabilities can be applied in an environmentally responsible and safe manner (Furey et al., 2020). With further development along these lines, we anticipate that strain *K. pneumoniae* strain Y1 (or its enzymes) could be harnessed for sustainable bioremediation of oily wastes without compromising public health or ecosystem safety.

## 5 Conclusion

In conclusion, this study demonstrates the remarkable oil-degrading capability of *K. pneumoniae* strain Y1 and its promising potential for application in treatment of oil-laden wastes. *K. pneumoniae* strain Y1 achieved rapid and efficient degradation of concentrated kitchen grease under optimized culturing conditions, outperforming many previously reported strains in terms of oil removal and COD reduction. Its broad substrate range and resilience to varying conditions underscore its value as a bioremediation agent for diverse oily pollutants. At the same time, recognizing that Y1 is a *K. pneumoniae* strain, we emphasize the importance of biosafety measures—such as cell immobilization and genetic containment strategies—to mitigate any risks associated with its use. With proper containment and engineering controls in place, the potent lipid-degradation activity of *K. pneumoniae* strain Y1 can be safely harnessed for environmental cleanup and wastewater treatment. This work therefore not only demonstrates Y1’s superior performance in breaking down fat-rich wastes, but also provides a framework for deploying such high-efficiency microbes in an effective and responsible manner for sustainable waste management.

## Data Availability

The data presented in this study are publicly available in figshare under the following DOI: 10.6084/m9.figshare.30847112.
